# CMTR-1 RNA methyltransferase mutations activate widespread expression of a dopaminergic neuron-specific mitochondrial complex I gene

**DOI:** 10.1016/j.cub.2024.04.079

**Published:** 2024-05-28

**Authors:** Joshua D. Meisel, Presli P. Wiesenthal, Vamsi K. Mootha, Gary Ruvkun

**Affiliations:** 1 Department of Molecular Biology, Massachusetts General Hospital, Boston, MA 02114, USA; 2 Harvard Medical School, Boston, MA 02115, USA; 3 Broad Institute, Cambridge, MA 02142, USA; 4 Howard Hughes Medical Institute, Massachusetts General Hospital, Boston, MA 02114, USA; 5 Lead contact

## Abstract

The mitochondrial proteome is comprised of approximately 1,100 proteins^1^, all but 12 of which are encoded by the nuclear genome in *C. elegans*. The expression of nuclear-encoded mitochondrial proteins varies widely across cell lineages and metabolic states^2–4^, but the factors that specify these programs are not known. Here we identify mutations in two nuclear-localized mRNA processing proteins, CMTR1/CMTR-1 and SRRT/ARS2/SRRT-1, which we show act via the same mechanism to rescue the mitochondrial complex I mutant *NDUFS2/gas-1(fc21)*. CMTR-1 is an FtsJ-family RNA methyltransferase that in mammals 2’-O-methylates the first nucleotide 3’ to the mRNA CAP to promote RNA stability and translation^5–8^. The mutations isolated in *cmtr-1* are dominant and lie exclusively in the regulatory G-patch domain. SRRT-1 is an RNA binding partner of the nuclear cap-binding complex and determines mRNA transcript fate^9^. We show that *cmtr-1* and *srrt-1* mutations activate embryonic expression of NDUFS2/*nduf-2*.2, a paralog of NDUFS2/*gas-1* normally expressed only in dopaminergic neurons, and that *nduf-2.2* is necessary for the complex I rescue by the *cmtr-1* G-patch mutant. Additionally, we find that loss of the *cmtr-1* G-patch domain cause ectopic localization of CMTR-1 protein to processing bodies (P-bodies), phase-separated organelles involved in mRNA storage and decay^10^. P-body localization of the G-patch mutant CMTR-1 contributes to the rescue of the hyperoxia sensitivity of the *NDUFS2/gas-1* mutant. This study suggests that mRNA methylation at P-bodies may control *nduf-2.2* gene expression, with broader implications for how the mitochondrial proteome is translationally remodeled in the face of tissue-specific metabolic requirements and stress.

## In Brief

Meisel et al. reveal that a *C. elegans* mitochondrial complex I mutant is rescued by mutations in the G-patch domain of the mRNA methyltransferase CMTR-1, which cause ectopic localization of the CMTR-1 protein to Processing bodies and widespread expression of the *NDUFS2* paralog *nduf-2.2* that is normally restricted to dopaminergic neurons.

## Graphical Abstract

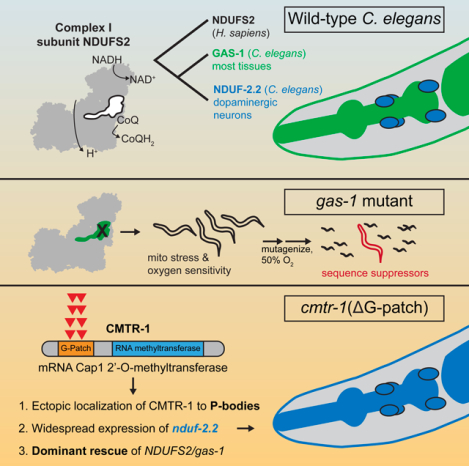

## RESULTS AND DISCUSSION

### CMTR-1 G-patch mutations rescue the mitochondrial *NDUFS2*/*gas-1(fc21)* mutant

In an effort to identify genetic regulators of the mitochondrial electron transport chain (ETC) across eukaryotes, we performed a forward genetic selection in *C. elegans* for mutations that rescue survival of the complex I mutant NDUFS2/*gas-1(fc21)*. NDUFS2 is a core subunit of complex I of the ETC that contributes to forming the quinone binding site^11–13^. The *fc21* allele encodes a hypomorphic R290K missense mutation that reduces complex I function to cause low broodsize and a moderate slowing of growth at 21% oxygen^14,15^. However at 50% oxygen – a condition that does not affect the growth of wild type animals – the *gas-1(fc21)* mutant arrests development at the L2 stage^16^. We randomly mutagenized *gas-1(fc21)* animals at the permissive 21% oxygen concentration, and then transferred F2 animals containing thousands of newly-induced mutations to the non-permissive 50% oxygen. Nearly all F2 animals arrested development at 50% oxygen, but rare animals that reached adulthood due to suppressor mutations that improved ETC function were selected, allowed to reproduce, retested for suppression of *gas-1(fc21)* hyperoxia sensitivity, and subjected to whole genome sequencing.

**Figure 1.**
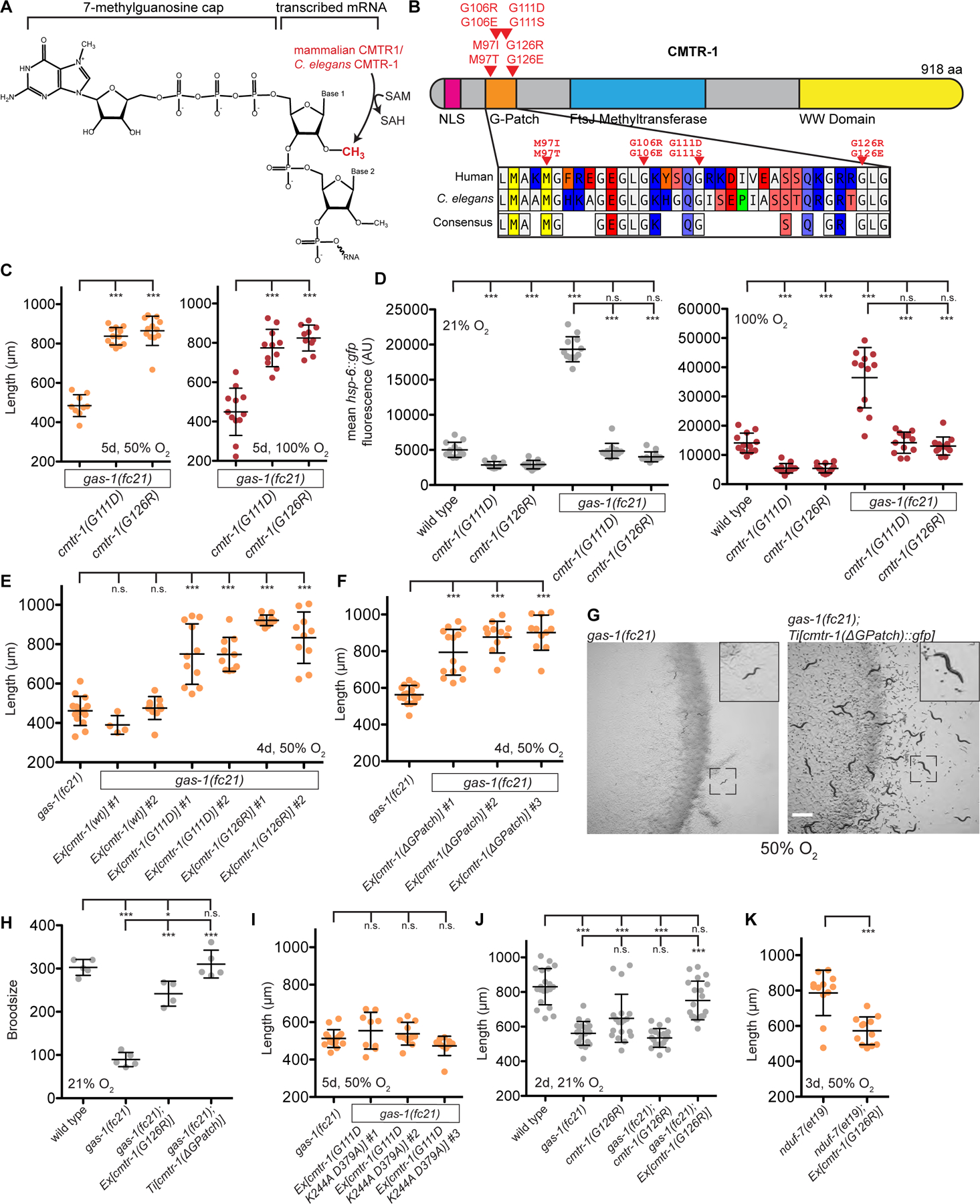
CMTR-1 G-patch mutations restore health of *NDUFS2/gas-1(fc21)* mutants A. CMTR1/CMTR-1 is a SAM-dependent RNA methyltransferase that has been shown in mammalian cells to 2’-O-methylate the first transcribed nucleotide of nuclear-encoded mRNAs. B. Mutations in *cmtr-1* that allow the *NDUFS2/gas-1(fc21)* mutant to survive hyperoxia are confined to conserved features of the G-patch domain^17^ (Pfam Signature PF01585). C. Growth of animals after 5 days exposure to 50% oxygen (left) or 5 days exposure to 100% oxygen followed by 3 days recovery at 21% oxygen (right). D. Mean intestinal fluorescence of the mitochondrial stress reporter *hsp-6::gfp* in L4 stage animals incubated at 21% or 100% oxygen for 1 day at 20°C. Exposure time = 100 ms, magnification = 69x, quantified from images in Figure S1B. E-F. Growth of animals following 4 days exposure to 50% oxygen. G. Images of animal growth and reproduction follow 4 days exposure to 50% oxygen. H. Total progeny produced from individual animals incubated at 21% oxygen. I. Growth of animals following 5 days exposure to 50% oxygen. J. Growth of animals following 2 days exposure to 21% oxygen. K. Growth of animals exposed to 3 days of 50% oxygen followed by recovery for 3 days at 21% oxygen. For all panels statistical significance was calculated using one-way ANOVA followed by Tukey’s Multiple Comparison Test. Error bars represent standard deviation. n.s. = not significant, * = p value <0.05, ** = p value <0.01, *** = p value <0.001. See also Figure S1 and Table S1.

From this genetic selection and genome sequencing, we identified 13 independent alleles of the gene *CMTR1*/*cmtr-1* which encodes a nuclear FtsJ-family RNA methyltransferase, the human orthologue of which has been shown biochemically to 2’-O-methylate the first transcribed nucleotide of mRNAs (Figure 1A). The 13 independent alleles we identified include 8 missense mutations (M97I, M97T, G106E, G106R, G111D, G111S, G126E, and G126R), all disrupting essential sequence features of the G-patch domain^17^ of CMTR-1 (Figure 1B & Table S1). Most of these mutations were isolated as heterozygotes in the strains that survived the selection, suggesting that these mutations confer a dominant phenotype. This G-patch domain, including the specific methionine and glycine residues substituted in the *cmtr-1 C. elegans* mutants, is conserved in mammalian CMTR1 (Figure 1B) and mediates an interaction with the RNA helicase DHX15/DDX-15^18,19^, but reports conflict about whether this interaction promotes or inhibits CMTR-1 methyltransferase activity. We confirmed that backcrossed screen isolates carrying mutations in the G-patch domain of CMTR-1 suppress the growth arrest of *gas-1(fc21)* at 50% and 100% oxygen (Figures 1C & S1A). *cmtr-1* G-patch mutations also completely suppressed induction of the *hsp-6::gfp* reporter for mitochondrial stress by *gas-1(fc21)* at 21% and 100% oxygen (Figures 1D & S1B), suggesting G-patch mutation results in healthier mitochondria in all oxygen tensions. Interestingly, 100% oxygen induces *hsp-6::gfp* expression in wild-type animals, and this response was also blunted in *cmtr-1* G-patch mutants (Figures 1D & S1B), suggesting that these G-patch mutations may be protective from oxygen-induced mitochondrial stress even in wild type.

CMTR-1 G-patch mutants were classically dominant with respect to *gas-1(fc21)* suppression, consistent with the heterozygous mutants that emerged from our selection (Figures S1C–D). Additionally, over-expression of transgenic *cmtr-1(G111D)* or *cmtr-1*-*(G126R)* in a wild-type *cmtr-1* background rescued *gas-1(fc21)* growth at 50% oxygen (Figure 1E), confirming a dominant genetic mode-of-action, and arguing against haploinsufficiency. In contrast, over expression of wild-type *cmtr-1* had no effect on *gas*-*1(fc21)* growth in high oxygen (Figure 1E), suggesting a gain-of-function activity of the mutant rather than a higher level of wild-type gene activity. Over-expression of *cmtr-1* harboring an in-frame deletion of the entire G-patch domain was also an excellent *gas*-*1(fc21)* suppressor at 50% oxygen (Figure 1F), pointing towards the G-patch domain being a negative regulator of CMTR-1 rescue activity. Indeed, single-copy insertion of *cmtr-1* harboring an in-frame G-patch deletion (with wild-type *cmtr-1* present at the endogenous locus) completely suppressed the growth arrest of *gas-1(fc21)* in hyperoxia and restored *gas-1(fc21)* brood size at 21% oxygen to wild-type levels (Figures 1G–H). Over-expression of G-patch mutant *cmtr-1(G111D)* with additional point mutations K244A and D379A that disrupt the catalytic site for RNA 2’-O-methylation^8,20^ did not rescue *gas-1(fc21)* in hyperoxia (Figure 1I), demonstrating that RNA methylation activity is required for G-patch mutant CMTR-1 rescue activity. Taken together, these results suggest that loss of the CMTR-1 G-patch domain leads to gain-of-function RNA methylation activity that rescues the *gas-1(fc21)* complex I mutant.

*gas-1(fc21)* mutants display a slight growth defect at 21% oxygen, which was curiously not rescued by *cmtr-1* mutants isolated from our screen (Figures 1J & S1E). However we observed that *cmtr-1* mutants have a recessive growth defect at 21% oxygen themselves, which may be responsible for the observed lack of rescue (Figure 1J). Indeed, over-expression of transgenic *cmtr-1(G126R)* with wild-type *cmtr-1* at the endogenous locus did rescue the slow growth of *gas-1(fc21)* at 21% oxygen (Figure 1J). Consistent with homozygous *cmtr-1* mutants having a recessive growth liability, heterozygous *cmtr-1* mutants rescued *gas-1(fc21)* growth rate in 50% oxygen better than homozygous mutants (Figure S1C), while homozygous *cmtr-1* mutations were a stronger suppressor of *hsp-6::gfp* induction (Figures 1D, S1B, and S1D). Additionally, over-expression of a *cmtr-1(G126R)* transgene rescued growth at 50% oxygen better than mutating *cmtr-1(G126R)* at the endogenous locus (Figure S1F). Taken together, these results demonstrate that *cmtr-1* G-patch loss rescues all aspects of the *gas-1(fc21)* mutant phenotype, and that endogenous *cmtr-1* G-patch loss slows wild-type growth.

We tested if *cmtr-1* G-patch mutations could rescue other complex I mutants, but transgenic over-expression of *cmtr-1(G126R)* did not improve growth of *NDUFS4/lpd-5(mg746)* null animals at 21% or 1% oxygen (Figure S1G) or *NDUFS7/nduf-7(et19)* hypomorph mutants at 21% or 50% oxygen (Figure 1K & S1H), and in fact tended to make these complex I mutants grow more slowly. Additionally, the *nduf-7(et19); cmtr-1(G111D)* double mutant displayed synthetic sickness, growing extremely slowly at 21% oxygen and producing an embryonic lethality phenotype, which neither the *nduf-7(et19)* nor *cmtr-1* single mutants displayed (Figure S1I). These results point to a rescuing activity of G-patch mutant CMTR-1 specific to the *gas-1(fc21)* mutant that may be detrimental in the context of other complex I lesions.

### G-patch mutant CMTR-1 is ectopically localized to Processing bodies

**Figure 2.**
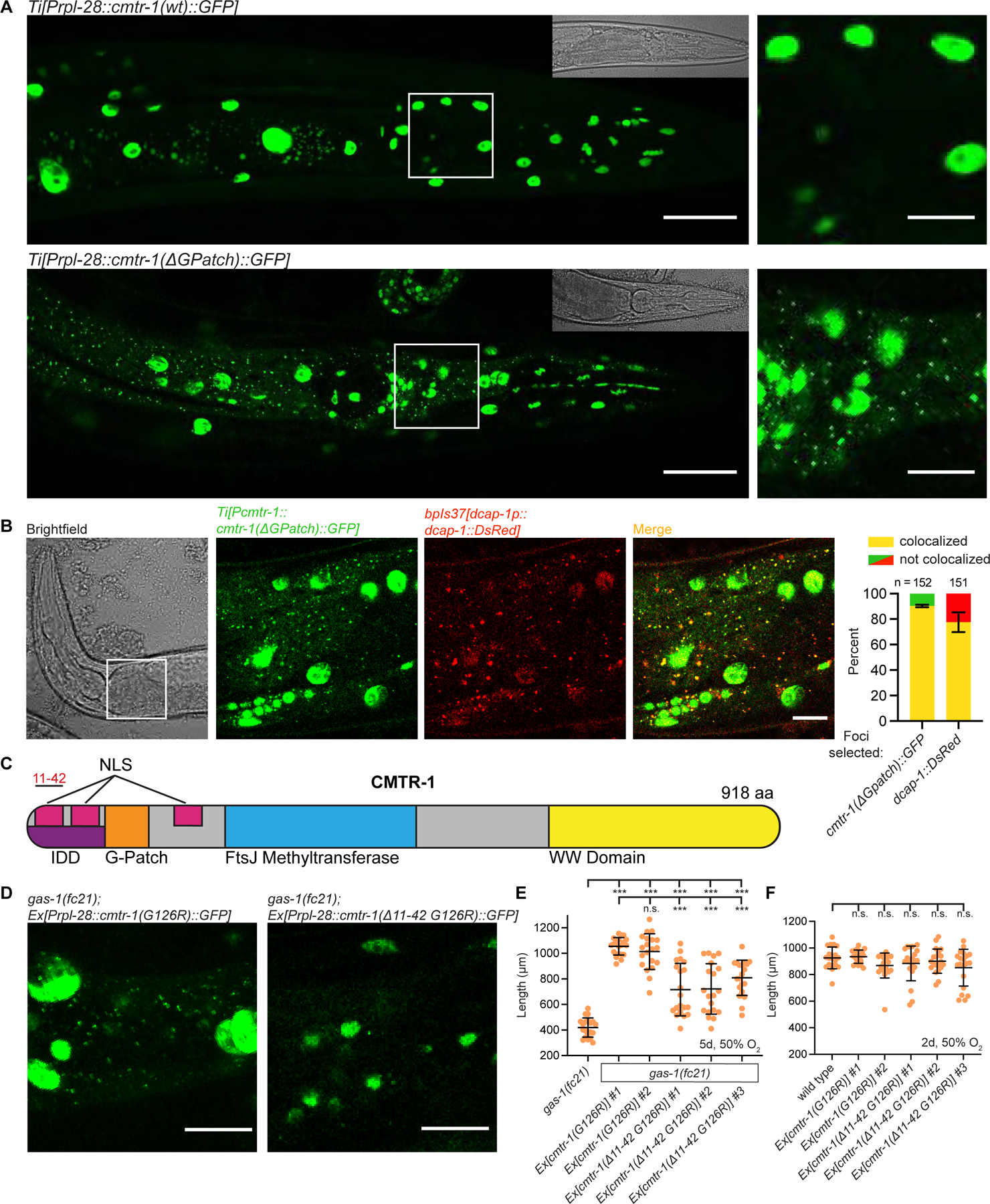
G-patch mutant CMTR-1::GFP is ectopically localized to P-bodies, which is necessary for its rescue activity A. Confocal microscopy of *cmtr-1(wt)::gfp* and *cmtr-1(ΔG-patch)::gfp* driven by the ribosomal promoter *Prpl-28*. Brightfield image is inset, white box corresponds to enlarged image on right. B. Confocal microscopy (left) and quantification (right) of *cmtr-1(ΔG-patch)::gfp* co-localization with the P-body marker *dcap-1::DsRed*. White box in brightfield image corresponds to fluorescent images on right. C. Bioinformatic analysis reveals three nuclear localization sequences in CMTR-1 and an N-terminal region predicted to form an intrinsically disordered domain. Deletion of amino acids 11–42 removes the first NLS and a portion of the IDD. D. Confocal microscopy of animals carrying extra-chromosomal arrays encoding G-patch mutant *cmtr-1(G126R)::gfp* or *cmtr-1(Δ11–42 G126R)::gfp*. E-F. Growth of animals following 5 days (E) or 2 days (F) exposure to 50% oxygen. Statistical significance was calculated using one-way ANOVA followed by Tukey’s Multiple Comparison Test. Error bars represent standard deviation. n.s. = not significant, *** = p value <0.001. See also Figure S2.

To understand how loss of the CMTR-1 G-patch domain activates CMTR-1 and confers *gas-1(fc21)* rescue activity, we tagged integrated single-copy *cmtr-1* transgenes with GFP to assess changes in expression or localization. We first confirmed that C-terminally tagged CMTR-1::GFP was functional, by showing that *cmtr-1(ΔG-patch)::gfp* rescued *gas-1(fc21)* growth arrest at 50% oxygen (Figure 1G). We used confocal microscopy to examine the expression pattern of wild-type CMTR-1, and as expected for an mRNA cap1 methyltransferase found it to be localized to all nuclei in the animal (Figure 2A). CMTR-1 carrying the G-patch deletion mutation that activates CMTR-1 activity also localized to nuclei, but, surprisingly, also localized to cytosolic foci in many cells of the animal (Figure 2A). These results were consistent when transgenes were driven by a strong ribosomal promoter (*Prpl-28*) or the endogenous *cmtr-1* promoter, arguing against the localization pattern being an artifact of over-expression (Figures 2A & S2A). Additionally, the *gas-1(fc21)* mutation has no effect on the localization of wild-type or G-patch mutant CMTR-1 (Figure S2B).

To determine the identity of the cytosolic foci, we introduced fluorescently-tagged proteins with known localization patterns into the *cmtr-1(ΔG-patch)::GFP* strain. *dcap-1::DsRed* is a marker of processing bodies (P-bodies)^21^ and co-localized with 90% of CMTR-1(ΔG-patch) foci (Figure 2B). Conversely, 80% of P-body foci contained *cmtr-1(ΔG-patch)::GFP* (Figure 2B), confirming these phase-separated condensates as sites of CMTR-1 ectopic localization if bearing a G-patch domain mutation. P-bodies are membraneless, ribonucleoprotein-based organelles involved in mRNA storage, silencing, and decay^10^.

We sought to determine whether nuclear or P-body localized CMTR-1(ΔG-patch) mediates *gas-1(fc21)* rescue activity, and so attempted to construct a *cmtr-1* G-patch mutant lacking the nuclear localization sequence. Surprisingly our *cmtr-1(Δ11–42 G126R)::gfp* strain maintained its nuclear localization pattern and instead showed dramatically decreased P-body localization (Figures 2C–D). Further bioinformatic analysis revealed three regions of CMTR-1 that may function as bipartite nuclear localization sequences (NLS), two of which were retained in our *cmtr-1(Δ11–42 G126R)* construct (Figures 2C & S2C), explaining its nuclear localization. Additionally, we found the N-terminal region deleted in the *cmtr-1(Δ11–42 G126R)* construct is predicted to lie in an intrinsically disordered domain (IDD) that may contribute to phase separation and P-body localization (Figures 2C & S2C). Notably, the predicted IDD and downstream NLS are both present in human CMTR1 (Figure S2C), suggesting P-body localization of mammalian CMTR1 may also occur if its G-patch activity is altered in particular physiological conditions.

We tested whether this ΔIDD *cmtr-1(Δ11–42 G126R)* derivative could rescue the *gas-1(fc21)* mutant growth arrest in hyperoxia and found that CMTR-1 lacking P-body expression had significantly reduced rescue activity (Figure 2E). As a control, extra-chromosomal arrays encoding *cmtr-1(G126R)::gfp* were localized to P-bodies and nuclei and rescued *gas-1(fc21)* growth arrest (Figures 2D–E), ruling out an effect of array mosaicism. Additionally, both sets of transgenes had no effect on growth rate in hyperoxia in a wild-type genetic background (Figure 2F). These data show that P-body localization of G-patch mutant CMTR-1 is partially necessary for its *gas-1* mutant complex I rescue activity. We do not exclude the possibility that G-patch mutant CMTR-1 localized to the dilute phase of the cytosol also has *gas-1* rescue activity.

### G-patch mutant CMTR-1 rescues *NDUFS2/gas-1(fc21)* by activating expression of its paralog NDUF-2.2

**Figure 3.**
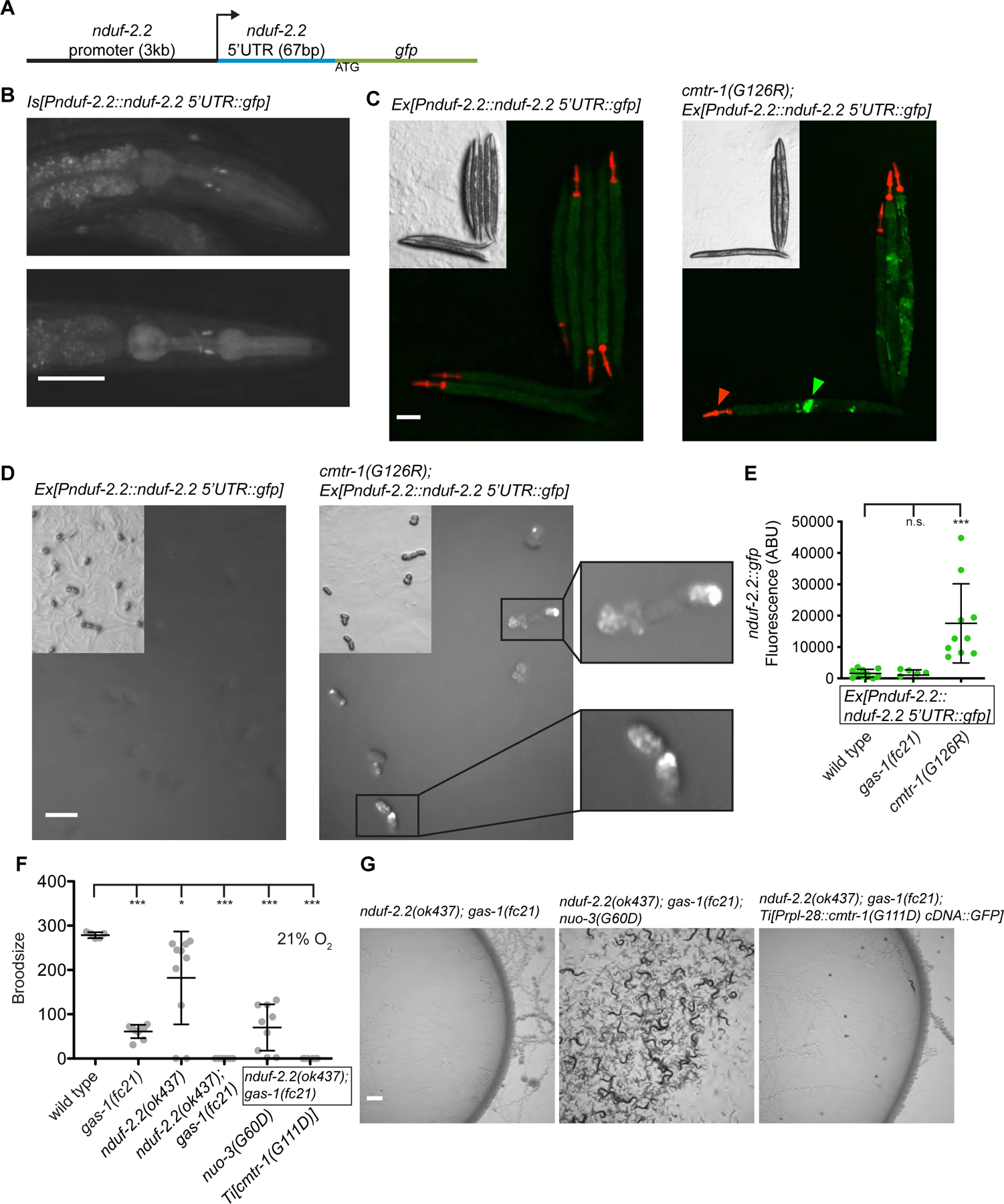
G-patch mutant CMTR-1 rescues *NDUFS2/gas-1(fc21)* by activating expression of the paralog NDUF-2.2 A. GFP reporter for *nduf-2.2* expression was constructed using 3 kb of upstream promoter sequence and 67 bp of the endogenous 5’UTR which is the target for cap1 2’-O-methylation by CMTR-1. B. *nduf-2.2::gfp* reporter in wild-type animals is weakly expressed in a subset of head neurons indicated by white arrows. Exposure time = 2 seconds, magnification = 100x. C. *nduf-2.2::gfp* reporter (green arrow) in wild-type or *cmtr-1(G126R)* adult animals. *myo-2::mCherry* co-injection marker (red arrow) is visible. Exposure time = 2 seconds, magnification = 40x. D. *nduf-2.2::gfp* reporter in wild-type and *cmtr-1(G126R)* embryos. White arrows indicate embryos expressing no GFP. Exposure time = 2 seconds, magnification = 90x. E. Quantification of *nduf-2.2::gfp* fluorescence in embryos from wild type and *cmtr-1(G126R)* (panel D) and *gas-1(fc21)* (not pictured). Plotted are maximum fluorescence values with background subtracted. F. Total progeny produced from individual animals incubated at 21% oxygen. G. Images of nematode growth at 21% oxygen after 1 generation. Statistical significance was calculated using t-test (E) or one-way ANOVA followed by Tukey’s Multiple Comparison Test (F). Error bars represent standard deviation. n.s. = not significant, * = p value <0.05, ** = p value <0.01, *** = p value <0.001. See also Figure S3.

Because P-bodies are sites of mRNA storage and translational control, we hypothesized that CMTR-1 may rescue *gas-1(fc21)* by activating translation of a particular transcript or set of transcripts. A strong candidate was *NDUFS2/nduf-2.2*, a paralog of *NDUFS2/gas-1* which is 96% identical after the mitochondrial targeting sequence^15^. For unknown reasons, gene duplication of the complex I NDUFS2 subunit has occurred many times in evolution and is present in diverse nematode species and helminths^22^. The *C. elegans gas-1(fc21)* R290K mutant sensitivity to volatile anesthetics is rescued by expression of *nduf-2.2* from the *gas-1* promoter but not the native *nduf-2.2* promoter, demonstrating that they are functionally equivalent complex I subunits, but that *nduf-2.2* may not be widely expressed^15^. In contrast to the lethal phenotype of a *gas-1* null mutant, *nduf-2.2* null mutant animals are viable with no growth defect, or anesthetic or oxygen sensitivity^23^, but do have axon regeneration defects^24,25^. We made a transgenic reporter for *nduf-2.2* using 3kb of upstream promoter and the endogenous 67 bp *nduf-2.2* 5’UTR (which could be methylated by CMTR-1 and/or subject to trans-splicing) fused to *gfp* (Figure 3A). In wild-type animals, we observed expression of *nduf-2.2* in dopaminergic neurons, as identified by co-localization with the dopamine transporter gene *dat-1::mCherry* (Figures 3B & S3A). When this *nduf-2.2* reporter gene with the *nduf-2.2* promoter and 5’UTR was introduced into the *cmtr-1* G-patch mutant, we observed radically expanded pattern of *nduf-2.2* expression in many cells of embryos and adult tissues (Figures 3C–E and S3B). No change in *nduf-2.2* expression was observed in the *gas-1(fc21)* background (Figure 3E), consistent with that mutant’s complex I deficiency. This data shows that the *cmtr-1* G-patch mutation activates *nduf-2.2* transcription or translation in additional cells and is consistent with the hypothesis that misexpression of the *nduf-2.2* paralog in the *cmtr-1* G-patch mutants is the molecular basis of the *gas-1(fc21)* mutant suppression.

To prove this hypothesis, we tested whether *nduf-2.2* was required for the rescue of *gas-1(fc21)* by a *cmtr-1* G-patch mutant. Although *nduf-2.2(ok437)* null mutants are viable and do not grow slowly, *gas-1(fc21)* is synthetic maternal effect lethal with *nduf-2.2* loss^23^. We constructed *gas-1(fc21); nduf-2.2(ok437)* double mutants with the use of a balancer chromosome for *gas-1* and confirmed that the double mutants which segregated were sterile (Figures 3F–G). This synthetic sterility was rescued by *nuo-3(G60D)*, an intra-complex suppressor mutation in complex I we have reported to boost complex I forward activity in the *gas-1(fc21)* mutant^16^ (Figures 3F–G). *cmtr-1* is a stronger *gas-1(fc21)* suppressor than *nuo-3(G60D)* with respect to many phenotypes including *hsp-6::gfp* attenuation, growth rate rescue, and hyperoxia resistance, but unlike *nuo-3(G60D)*, the G-patch mutant *cmtr-1* was not able to rescue the synthetic lethality of *gas-1(fc21)* and *nduf-2.2(ok437)* (Figures 3F–G). This data proves that *cmtr-1* rescue of *gas-1(fc21)* is dependent on the paralog *NDUFS2/nduf-2.2*.

The NDUFS2 duplication is present in all *Caenorhabditis* species as well as a diverse subset of nematode species (e.g. *Ascaris suum*, *Brugia malayi*, *Onchocerca volvulus*) separated by hundreds of millions of years of nematode evolution^22^, and specific amino acid differences between GAS-1 and NDUF-2.2 are highly conserved in these nematode species (Figures S3C–D). This data, along with the endogenous expression in dopaminergic neurons and neuronal phenotypes of *nduf-2–2*, raises the question of what the functional differences are between *gas-1* and *nduf-2.2*, and whether they might be relevant for the rescue by *cmtr-1* G-patch mutants. It has been hypothesized that paralogs *nduf-2.2* and *sdha-2* may produce alternate ETC complexes I and II (respectively) that use rhodoquinone (RQ) to couple forward activity of complex I to reverse activity of complex II in states of hypoxia^22^. RQ synthesis is dependent on tryptophan degradation and modification by the kynurenine pathway; a *kynu-1* mutation that disables a step in rhodoquinone synthesis disrupts the production of RQ^26^. We introduced *kynu-1(tm4924)* into the *gas-1(fc21)* mutant and found that G-patch mutant *cmtr-1* was still able to rescue the growth arrest in hyperoxia (Figure S3E), demonstrating that rescue does not require RQ and therefore it is unlikely complex II runs in reverse in these mutants. Instead, we hypothesize that forward flow through the ETC is restored by *cmtr-1* mutation and *nduf-2.2* expression in the *gas-1(fc21)* mutants.

### Mutation of the RNA binding protein Serrate rescues *NDUFS2/gas-1(fc21)*

**Figure 4.**
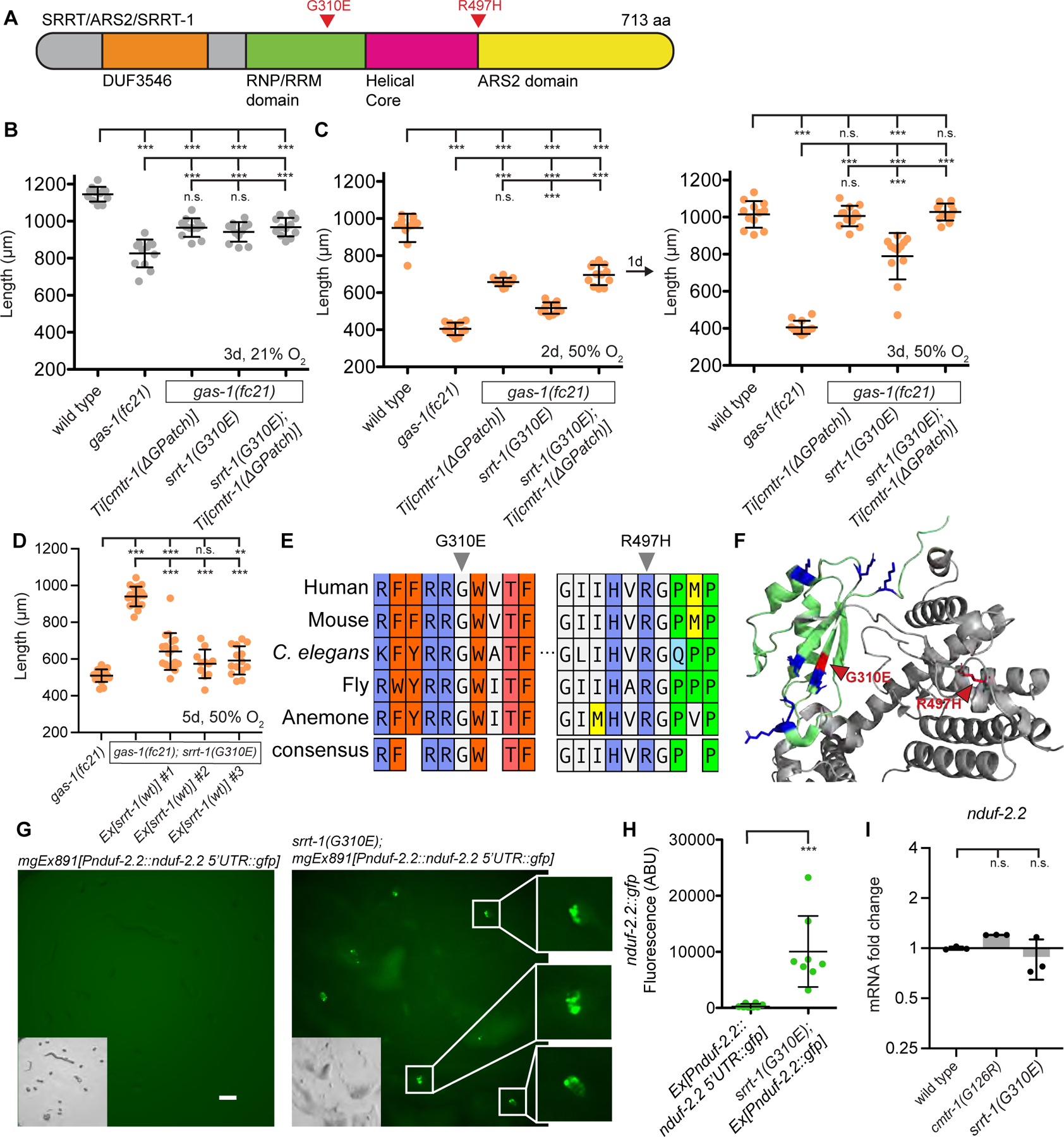
Mutation of the RNA binding protein Serrate activates *nduf-2.2* and rescues *gas-1(fc21)* A. Domain structure of RNA binding protein Serrate. B. Growth of animals following 3 days exposure to 21% oxygen. C. Growth of animals following 2 days (left) or 3 days (right) exposure to 50% oxygen. D. Growth of animals following 5 days exposure to 50% oxygen. E. Multiple sequence alignment of *SRRT/srrt-1* homologs from animals made with ClustalW. Labelled residues correspond to suppressor mutations isolated in *C. elegans srrt-1*. F. Crystal structure of Human SRRT/ARS2 (PDB: 6F7J^27^). Highlighted in green is the RNA-binding RRM domain. Blue residues correspond to positively charged amino acids; red residues correspond to mutations isolated in this study. G. *nduf-2.2::gfp* reporter in wild-type and *srrt-1(G310E)* embryos. White arrows indicate embryos expressing no GFP. Exposure time = 1 second, magnification = 90x. H. Quantification of GFP fluorescence in embryos from panel G. Plotted are maximum fluorescence values with background subtracted. I. Quantitative real-time PCR of *nduf-2.2* mRNA normalized to the housekeeping gene *rps-23*. For all panels statistical significance was calculated using one-way ANOVA followed by Tukey’s Multiple Comparison Test. Error bars represent standard deviation. n.s. = not significant, * = p value <0.05, ** = p value <0.01, *** = p value <0.001. See also Figure S4.

To further understand the mechanism by which CMTR-1 localization at P-bodies may activate *NDUFS2/nduf-2.2* expression, we returned to our *gas-1(fc21)* suppressor screen and searched for additional lesions in mRNA processing genes in the sequenced isolates that did not contain *cmtr-1* mutations. We identified three independent alleles of Serrate*/SRRT/ARS2/srrt-1* that encode G310E or R497H missense mutations and confer a recessive *gas-1(fc21)* rescue phenotype (Figure 4A & Table S1). Serrate is a nuclear RNA binding protein that interacts with single stranded RNA and the cap binding complex in plants and animals to target transcribed RNAs to distinct cellular fates^27^. We used CRISPR-Cas9 to introduce the *srrt-1(G310E)* mutation into a clean genetic background and confirmed that *srrt-1(G310E)* rescued *gas-1(fc21)* growth rate at 21% and 50% oxygen (Figures 4B–C). Over-expression of wild-type *srrt-1* rescued the *srrt-1(G310E)* mutant, resulting in slower growth in a *gas-1(fc21)* mutant background at 50% oxygen (Figure 4D), confirming the reduction-of-function nature of the *srrt-1(G310E)* allele. Over-expression of *srrt-1(wt)* had no effect on growth at 50% oxygen in a wild-type background (Figure S4A). Both *SRRT/srrt-1* mutations isolated in our screen lie in highly conserved residues present in all metazoa (Figure 4E). By introducing a negative charge the G310E mutation may disrupt the RNA recognition motif (RRM) domain, a basic patch of positively charged Arg and Lys residues that interacts with single stranded RNA (Figure 4F)^27^.

To determine if *srrt-1* and *cmtr-1* act via a shared mechanism to rescue *gas-1(fc21)* mutants, we constructed the *srrt(G310E); cmtr-1(G-patch); gas-1(fc21)* triple mutant and observed that *cmtr-1* and *srrt-1* were not additive for *gas-1(fc21)* rescue at 21% or 50% oxygen (Figures 4B–C), consistent with both genes acting in the same genetic pathway. In contrast, *nuo-3(G60D)* and *cmtr-1(G111D)* were additive for *gas-1(fc21)* suppression (Figure S4B), indicating that improvement of the *cmtr-1* rescue of *gas-1(fc21)* is possible. Importantly, like the *cmtr-1(G-patch)* mutant, the *srrt-1(G310E)* mutation induced expression of the *nduf-2.2::gfp* reporter in embryos (Figures 4G–H), confirming *cmtr-1* and *srrt-1* both activate *nduf-2.2* expression. We performed qPCR using primers specific for *nduf-2.2* (Figure S4C) and found that neither *cmtr-1(G126R)* nor *srrt-1(G310E)* mutation caused a significant increase in *nduf-2.2* mRNA (Figure 4I), despite a dramatic increase in embryonic *nduf-2.2::gfp* expression. Notably, single-cell transcriptomics from wild-type embryos detected *nduf-2.2* mRNA in 94/409 cells^28^, consistent with a post-transcriptional regulatory mechanism. Taken together these results strongly suggest that mutations in *cmtr-1* and *srrt-1* rescue *gas-1(fc21)* by increasing the translation of *nduf-2.2* mRNA.

We asked if the *srrt-1* suppressor mutation acts directly through CMTR-1 by affecting its subcellular localization. However *srrt-1(G310E)* did not alter the wild-type *cmtr-1::gfp* localization pattern which remained exclusively nuclear (Figure S4D), showing that *srrt-1(G310E)* does not rescue *gas-1(fc21)* by causing ectopic localization of CMTR-1 to P-bodies. Additionally, *srrt-1(G310E)* did not alter the mutant *cmtr-1(ΔG-patch)::gfp* localization to nuclei and P-body foci, demonstrating that the *srrt-1* mutation does not act by disrupting P-body condensates (Figure S4E). Instead, we hypothesize that *srrt-1* mutation alters the localization of a specific mRNA transcript such as *nduf-2.2*, resulting in a fate equivalent to hyperactive RNA methylation by G-patch mutant CMTR-1. For example, SRRT-1 protein may normally traffic *nduf-2.2* mRNA to P-bodies, thus repressing its translation.

## Conclusions

Post-transcriptional chemical modification of RNA molecules is widespread, molecularly diverse, and confers dramatic impacts on gene expression^29,30^. CMTR-1 and cap1 methylation of mRNAs have not been implicated in playing a regulatory role in gene expression but rather are thought to be a feature of the 5’ mRNA cap on essentially all eukaryotic mRNAs^31^. In this study we show that loss of the highly conserved regulatory G-patch domain of CMTR-1 causes ectopic localization of the CMTR-1 protein to P-bodies and activation of the duplicated *NDUFS2/gas-1* paralog *NDUFS2*/*nduf-2.2* in many cells of the embryo, which in turn rescues *NDUFS2/gas-1(fc21)* complex I mutants from the toxicity of high oxygen. These results implicate a highly specific target mRNA for methylation by the CMTR-1 G-patch mutant and are generally consistent with human cell culture experiments that showed transgenic expression of G-patch mutant CMTR-1 caused no transcriptomic changes but rather increased translation of certain mRNAs^18^.

*cmtr-1* is one of five *C. elegans* members of the ftsJ RNA methyltransferase gene family that is conserved across the Tree of Life. Our genetic analysis generated gain-of function mutations that disrupt the G-patch domain of CMTR-1, one of 14 G-patch domain proteins in *C. elegans*, many of which bind RNA. CMTR-1 is the only ftsJ homolog among the five with a G-patch domain, which is a universal feature of animal CMTR1 orthologues. The other four *C. elegans* ftsJ RNA methyltransferase genes are: *cmtr-*2, an animal-specific ftsJ domain protein that methylates the second transcribed nucleotide of mRNAs^32^, and three other ftsJ genes (F45G2.9, H06I04.3, and R74.7) that have clear bacterial and archaeal orthologues and methylate their targets at RNA stem-loop structures^33,34^. Salient to our discovery of CMTR-1 regulation of mitochondrial electron transport gene expression, F45G2.9 is the orthologue of the mitochondrial-localized mammalian MRM2 that methylates the mitochondrial 16S ribosomal RNA. CMTR-1 may also have more RNA substrates in addition to its biochemical assignment as a cap1 methyltransferease, as deletion of *cmtr-2* in *C. elegans* did not affect levels of cap2 methylation^35^. Our study suggests the cap1 methylation and/or other RNA methylations mediated by CMTR-1 could be a rate-limiting step in the translation of particular target mRNAs rather than a constitutive feature of all mRNAs.

While the CMTR-1 G-patch mutant suppression of *NDUFS2*/*gas-1(fc21)* oxygen-sensitivity by expression of an NDUFS2 paralog is a simple single target gene mechanism, the implications for translational regulation of gene expression at P-bodies are profound. P-bodies were originally thought to be sites of RNA decay based on the presence of exonucleases and decapping enzymes, but subsequent evidence has shown that mRNAs in P-bodies are merely silenced and can reenter the cytosol and be translated^10^. Internal (non-cap) m^6^A mRNA methylation by YTHDF2 can target mRNAs to P-bodies^36^, but what modifications control the fate of P-body-localized mRNA remain unknown. We propose that CMTR1/CMTR-1-mediated RNA methylation may be an important regulatory mark that determines whether mRNAs at P-bodies initiate translation. In this study we focus on expression of *NDUFS2/nduf-2.2*, a subunit of complex I of the mitochondrial ETC. Future studies will characterize the full set of genes activated by mutant CMTR-1 and SRRT-1, and whether they belong to a shared biological process. We note that P-bodies are physically associated with mitochondria^37^ and that during glucose starvation mitochondrial transcripts are upregulated and stored in P-bodies but not decayed^38^.

Mitochondrial proteomes vary across developmental and metabolic states^3,4^, and the cytosolic and mitochondrial translational machinery are coupled in the face of increased demand for respiratory metabolism^39^, implicating a post-transcriptional regulatory mechanism. Indeed, nuclear mRNA splicing factors (e.g. U1 snRNP) were identified in a genome-wide CRISPR screen for gene disruptions that promote the shift to oxidative phosphorylation^40^. Our screen identified CMTR-1 and SRRT-1 as two more nuclear mRNA factors that may affect translation of nuclear-encoded mitochondrial proteins, and tantalizingly SRRT interacts with the U1 snRNP, promoting its loading onto most transcripts^9^. Thus our recessive mutations in SRRT may phenocopy the disruption of U1 snRNP activity. Whether mutations in SRRT also affect cap1 methylation or localization of certain mRNAs to P-bodies is an area of future study.

The *NDUFS2/nduf-2.2* paralog requires either a *cmtr-1* activating mutation or *srrt-1* loss-of-function mutation to be widely expressed, but we observed normal physiological expression of *nduf-2.2* in a subset of neurons that includes the dopaminergic neurons. This is consistent with neuronal phenotypes of the *nduf-2.2* mutant, which displays axon regeneration defects in the PLM mechanosensory neurons that express a D1-like dopamine receptor. Dopaminergic neurons experience a high burden of oxidative stress^41^ and lowered complex I activity may underlie the death of dopaminergic neurons in Parkinson’s disease^42–44^. The amino acid differences between GAS-1 and NDUF-2.2 are highly conserved across the taxonomy of nematodes and platyhelminths. We hypothesize that the duplicated NDUF-2.2 subunit may produce a specialized complex I adapted to the metabolic state of dopaminergic neurons. The G-patch of *C. elegans* CMTR-1 is required to negatively regulate expression of *nduf-2.2* in cells other than dopaminergic neurons. Future work will investigate whether the activities of CMTR-1 or SRRT-1 are altered in specific cells or under particular conditions (for example, oxygen tension or high NADH levels) and may play a physiological role in the expression of *nduf-2-2*, potentially delineating mechanisms underlying the variation in the mitochondrial proteome across cell lineages or metabolic states.

## STAR Methods

### RESOURCE AVAILABILITY

#### Lead contact

Further information and requests for resources and reagents should be directed to and will be fulfilled by the lead contact, Gary Ruvkun (ruvkun@molbio.mgh.harvard.edu).

#### Materials availability

*C. elegans* strains and plasmids generated in this study are available upon request from the lead contact.

#### Data and code availability

- All data reported in this paper will be shared by the lead contact upon request.
- This paper does not report original code.
- Any additional information required to reanalyze the data reported in this paper is available from the lead contact upon request.

### EXPERIMENTAL MODELS AND STUDY PARTICIPANT DETAILS

#### Strain maintenance and generation

*C. elegans* were propagated on NGM plates seeded with *E. coli* strain OP50^45^. A complete list of strains used in this study can be found in the Key Resources Table. Some strains were provided by the CGC, which is funded by NIH Office of Research Infrastructure Programs (P40 OD010440). To generate mutants with CRISPR/Cas9, 30 pmol *S. pyogenes* Cas9 (IDT) was injected into *C. elegans* gonads along with 90 pmol tracrRNA (IDT), 95 pmol crRNA (IDT), ssODN repair template (when applicable), and 40 ng/μl PRF4::*rol-6(su1006)* plasmid was used as a marker of successful injections^46^. To generate transgenic animals carrying extra-chromosomal arrays, a mix consisting of 50 ng/μl plasmid DNA of interest and 50 ng/μl plasmid DNA containing *ofm-1::gfp* was injected into *C. elegans* P0 gonads. F1 progeny displaying the co-injection marker were singled to new plates and screened for lines in which the array was inherited by the F2 generation; at least three independent lines were generated for each construct. To generate animals carrying single-copy integrated transgenes, a plasmid mix consisting of 50 ng/μl *Mos1* transposase, 2 ng/μl *myo-2::mCherry*, 2 ng/μl *myo-3::mCherry*, and 12 ng/μl *miniMos* transgene was injected into *unc-119(ed3)* mutant *C. elegans*^47^. Injected P0s were immediately placed at 25°C to avoid transgene silencing, and integrated non-unc non-red transgenic animals were confirmed by backcrossing and Mendelian segregation.

### METHOD DETAILS

#### Genetic screens and sequence analysis

To screen for genetic suppressors of *gas-1(fc21)* in hyperoxia thousands of L4 animals were exposed to 47 mM EMS (ethyl methanesulfonate) (Sigma M0880) for four hours while rocking. Animals were then washed twice with M9 buffer and allowed to recover on standard NGM plates. F1 animals were bleach prepped as described above to generate a synchronized L1 stage population of mutagenized F2 animals, which were then dropped onto standard NGM plates at 50% oxygen. Plates were checked daily and F2 individuals capable of growing to adulthood were transferred onto new plates. Fertile isolates were retested using F3 or F4 progeny to confirm their phenotype and then genomic DNA for whole genome sequencing was isolated using Gentra Puregene Tissue Kit (Qiagen 158667).

To identify candidate suppressor mutations in screen isolates we sheared genomic DNA using a Covaris S2 sonicator and prepared libraries using the NEBNext DNA library prep kit for Illumina. Libraries with unique barcodes were quantified using the Qubit dsDNA HS Assay Kit (Life Technologies Q32851) and pooled in sets of 24 and sequenced using Illumina HiSeq^48^. Raw FASTQ files were analyzed on the Galaxy platform (usegalaxy.org) with the following workflow: TrimGalore! to trim reads, Map with BWA to align reads to the *C. elegans* reference genome, MiModD to call variants, and SnpEff to identify mutations that may affect protein function. Lists of protein-altering mutations from each suppressor strain were then compared to identify genes with multiple mutant alleles. These candidate genes were then verified using targeted CRISPR/Cas9-based editing.

For protein sequence comparisons, homologs were identified using BlastP and, in the case of NDUFS2, from https://caenorhabditis.org/^49^ and alignments made with ClustalW. For annotation of *C. elegans* CMTR-1 and human CMTR1 sequence features, predicted nuclear localization signals were identified using cNLS mapper^50^ and intrinsically disordered domains were identified using both DEPICTER^51^ and PrDOS^52^.

#### Growth and fluorescent reporter assays

To measure *C. elegans* growth and development crowded plates of gravid animals were washed into tubes in M9 buffer [3 g KH_2_PO_4_, 6 g Na_2_HPO_4_, 5 g NaCl, 1 ml 1 M MgSO_4_, H_2_O to 1 liter] and incubated with 20% bleach and 10% 5M KOH for 5 minutes while vortexing. The resulting embryos were washed 3x in M9 buffer and allowed to hatch overnight while rocking in M9. The following day arrested L1 animals were dropped onto *E. coli* OP50 plates and incubated at 20°C. For assays in hyperoxia (50% or 100% oxygen) plates were sealed in a modular chamber (Stemcell Technologies #27310) and flushed for 3 minutes with either a 50:50 mixture of oxygen and nitrogen, or with pure oxygen gas. For assays in hypoxia (1% oxygen), animals were incubated in a Hypoxic *in vitro* cabinet (Coy Laboratory Products, Inc) at room temperature. To measure animal length, images were acquired using a ZEISS Axio Zoom V16 microscope with ZEN PRO software and the midline of individual animals was quantified in FIJI software. Brood size measurements were made by transferring adult *C. elegans* to new plates daily and counting all progeny generated over the course of egg-laying adulthood.

To measure *hsp-6::gfp* and *nduf-2.2::gfp* fluorescence, animals were mounted on agar pads, immobilized in sodium azide, and imaged at 70x magnification using a ZEISS Axio Zoom V16 microscope with ZEN PRO software. Fluorescent images were quantified by calculating the mean fluorescence along the midline of the intestine (for *hsp-6::gfp)* or by calculating the maximum fluorescence in embryos (for *nduf-2.2::gfp)* using FIJI software. To visualize *cmtr-1::gfp* localization patterns, images were acquired with a Nikon A1R confocal microscope, using a 60X/1.49 NA oil objective using 488nm excitation at 290nm/pixel. Worms were immobilized on 10% agarose pads with 0.3 μl of 0.1 μm diameter polystyrene microspheres (Polysciences 00876-15, 2.5% w/v suspension). To quantify colocalization of *cmtr-1(ΔG-patch)::gfp* and *dcap-1::DsRed*, green or red foci from three independent animals were blindly selected and the presence or absence of foci in the other channel was scored.

#### Quantitative PCR

To measure *nduf-2.2* mRNA levels by qPCR, total RNA was isolated from mixed stage animals using TRIzol Reagent (ThermoFisher 15596026). RNA was DNase treated using DNA-free Kit (Invitrogen AM1906) and cDNA was synthesized by the ProtoScript II First Strand cDNA Synthesis Kit (NEB E6560). Quantitative real-time PCR was performed using iQ SYBR Green Supermix (Biorad) on a BIORAD CFX Real-Time System. Three biological replicates were analyzed for each genotype, and three technical replicates per sample were performed in each qPCR run. Negative controls included (1) cDNA samples synthesized without reverse transcriptase and (2) cDNA synthesized from *nduf-2.2* deletion mutants, confirming that the *nduf-2.2* primers were specific. Delta Cq values were normalized to the housekeeping gene *rps-23*. All primer sequences are available in the Key Resources Table.

### QUANTIFICATION AND STATISTICAL ANALYSYS

All statistical analyses were performed using GraphPad Prism software. Statistical tests are detailed in the Figure Legends. Typically, statistical significance was calculated using one-way ANOVA followed by correction for multiple hypothesis testing. Error bars represent standard deviation and ‘n’ refers to the number of animals tested in a single experiment. n.s. = not significant, * = p value <0.05, ** = p value <0.01, *** = p value <0.001.

## Supplementary Material

2

## Figures and Tables

**Table:** 

REAGENT or RESOURCE	SOURCE	IDENTIFIER
Experimental models: Organisms/strains		
*C. elegans* strain CW152*: gas-1(fc21) X +22.9*	CGC	CW152
*C. elegans* strain GR3408*: nduf-7(et19) I*	Ruvkun lab^16^, derived from QC134 (CGC)	GR3408
*C. elegans* strain GR3414*: hsp-6::gfp; gas-1(fc21)*	Ruvkun Lab^16^	GR3414
*C. elegans* strain GR3489*: cmtr-1(mg780[G126R]); gas-1(fc21)*	This study	GR3489
*C. elegans* strain GR3490*: cmtr-1(mg779[G111D]); gas-1(fc21)*	This study	GR3490
*C. elegans* strain GR3491*: cmtr-1(mg779[G111D]); hsp-6::gfp; gas-1(fc21)*	This study	GR3491
*C. elegans* strain GR3492*: cmtr-1(mg779[G111D]); hsp-6::gfp*	This study	GR3492
*C. elegans* strain GR3493*: cmtr-1(mg780[G126R]); hsp-6::gfp*	This study	GR3493
*C. elegans* strain GR3494*: cmtr-1(mg780[G126R]); hsp-6::gfp; gas-1(fc21)*	This study	GR3494
*C. elegans* strain GR3495*: gas-1(fc21); mgEx878[Prpl-28::cmtr-1 cDNA::GFP + myo-2::mCherry]*	This study	GR3495
*C. elegans* strain GR3496*: gas-1(fc21); mgEx879[Prpl-28::cmtr-1 cDNA::GFP + myo-2::mCherry]*	This study	GR3496
*C. elegans* strain GR3497*: gas-1(fc21); mgEx880[Prpl-28::cmtr-1(G111D) cDNA::GFP + myo-2::mCherry]*	This study	GR3497
*C. elegans* strain GR3498*: gas-1(fc21); mgEx881[Prpl-28::cmtr-1(G111D) cDNA::GFP + myo-2::mCherry]*	This study	GR3498
*C. elegans* strain GR3499*: gas-1(fc21); mgEx882[Prpl-28::cmtr-1(G126R) cDNA::GFP + myo-2::mCherry]*	This study	GR3499
*C. elegans* strain GR3500*: gas-1(fc21); mgEx883[Prpl-28::cmtr-1(G126R) cDNA::GFP + myo-2::mCherry]*	This study	GR3500
*C. elegans* strain GR3501*: gas-1(fc21); mgEx884[Prpl-28::cmtr-1(DeltaG-Patch) cDNA::GFP + myo-2::mCherry]*	This study	GR3501
*C. elegans* strain GR3502*: gas-1(fc21); mgEx885[Prpl-28::cmtr-1(DeltaG-Patch) cDNA::GFP + myo-2::mCherry]*	This study	GR3502
*C. elegans* strain GR3503*: gas-1(fc21); mgEx886[Prpl-28::cmtr-1(DeltaG-Patch) cDNA::GFP + myo-2::mCherry]*	This study	GR3503
*C. elegans* strain GR3504*: gas-1(fc21); mgTi63[Prpl-28::cmtr-1(DeltaG-Patch) cDNA::GFP]*	This study	GR3504
*C. elegans* strain GR3505*: gas-1(fc21); mgEx887[Prpl-28::cmtr-1(G111D K244A D379A) cDNA::GFP + myo-2::mCherry]*	This study	GR3505
*C. elegans* strain GR3506*: gas-1(fc21); mgEx888[Prpl-28::cmtr-1(G111D K244A D379A) cDNA::GFP + myo-2::mCherry]*	This study	GR3506
*C. elegans* strain GR3507*: gas-1(fc21); mgEx889[Prpl-28::cmtr-1(G111D K244A D379A) cDNA::GFP + myo-2::mCherry]*	This study	GR3507
*C. elegans* strain GR3508*: cmtr-1(mg780[G126R]) II*	This study	GR3508
*C. elegans* strain GR3509*: nduf-7(et19); mgEx882[Prpl-28::cmtr-1(G126R) cDNA::GFP + myo-2::mCherry]*	This study	GR3509
*C. elegans* strain SJ4100*: zcIs13[hsp-6::GFP] V*	CGC	SJ4100
*C. elegans* strain GR3513*: unc-119(ed3) III; mgTi64[Prpl-28::cmtr-1 cDNA::GFP]*	This study	GR3513
*C. elegans* strain GR3514*: unc-119(ed3) III; mgTi63[Prpl-28::cmtr-1(DeltaG-Patch) cDNA::GFP]*	This study	GR3514
*C. elegans* strain GR3515*: unc-119(ed3) III; mgTi65[Pcmtr-1::cmtr-1(no G-patch) cDNA::GFP + unc-119(+)]; bpIs37[dcap-1p::dcap-1::DsRed + rol-6(su1006)]*	This study	GR3515
*C. elegans* strain GR3516*: gas-1(fc21); mgEx890[Prpl-28::cmtr-1(Delta11–42 G126R) cDNA::GFP + myo-2::mCherry]*	This study	GR3516
*C. elegans* strain GR3519*: mgIs86[Pnduf-2.2(3 kb)::gfp + myo-2::mCherry] 8xBC*	This study	GR3519
*C. elegans* strain GR3520*: mgEx891[Pnduf-2.2(3 kb)::gfp + myo-2::mCherry]*	This study	GR3520
*C. elegans* strain GR3521*: cmtr-1(mg780[G126R]); mgEx891[Pnduf-2.2(3 kb)::gfp + myo-2::mCherry]*	This study	GR3521
*C. elegans* strain VC393*: nduf-2.2(ok437) III*	CGC	VC393
*C. elegans* strain GR3522*: nduf-2.2(ok437); gas-1(fc21)/tmC24[F23D12.4(tmIs1233) unc-9(tm9718)]*	This study	GR3522
*C. elegans* strain GR3523*: nduf-2.2(ok437); nuo-3(mg748[G60D]); gas-1(fc21)/tmC24[F23D12.4(tmIs1233) unc-9(tm9718)]*	This study	GR3523
*C. elegans* strain GR3524*: nduf-2.2(ok437) mgTi67[Prpl-28::cmtr-1(G111D) cDNA::GFP]; gas-1(fc21)/tmC24[F23D12.4(tmIs1233) unc-9(tm9718)]*	This study	GR3524
*C. elegans* strain GR3528*: srrt-1(mg781[G310E]); gas-1(fc21)*	This study	GR3528
*C. elegans* strain GR3529*: srrt-1(mg781[G310E]); mgTi63[Prpl-28::cmtr-1(DeltaG-Patch) cDNA::GFP]; gas-1(fc21)*	This study	GR3529
*C. elegans* strain GR3446*: gas-1(fc21); mgEx869[Prpl-28::nuo-3(G60D) cDNA + ofm-1::gfp]*	Ruvkun Lab^16^	GR3446
*C. elegans* strain GR3530*: gas-1(fc21); mgTi68[Prpl-28::cmtr-1(G111D) cDNA::GFP]*	This study	GR3530
*C. elegans* strain GR3531*: gas-1(fc21); mgTi68[Prpl-28::cmtr-1(G111D) cDNA::GFP]; mgEx869[Prpl-28::nuo-3(G60D) cDNA + ofm-1::gfp]*	This study	GR3531
*C. elegans* strain GR3532*: srrt-1(mg781[G310E]); mgEx891[Pnduf-2.2(3 kb)::gfp + myo-2::mCherry]*	This study	GR3532
*C. elegans* strain GR3406*: lpd-5(mg746[354bp DEL])/hT2 I*	Ruvkun Lab^16^	GR3406
*C. elegans* strain GR3510*: lpd-5(mg746[354bp DEL])/hT2; mgEx882[Prpl-28::cmtr-1(G126R) cDNA::GFP + myo-2::mCherry]*	This study	GR3510
*C. elegans* strain GR3511*: cmtr-1(mg779[G111D]) II*	This study	GR3511
*C. elegans* strain GR3512*: nduf-7(et19); cmtr-1(mg779[G111D])*	This study	GR3512
*C. elegans* strain GR3517*: unc-119(ed3) III; mgTi66[Pcmtr-1::cmtr-1 cDNA::GFP + unc-119(+)]*	This study	GR3517
*C. elegans* strain GR3518*: unc-119(ed3) III; mgTi65[Pcmtr-1::cmtr-1(no G-patch) cDNA::GFP + unc-119(+)]*	This study	GR3518
*C. elegans* strain GR3525*: otIs181[Pdat-1::mCherry; Pttx-3::mCherry] III; mgIs86[Pnduf-2.2(3 kb)::gfp + myo-2::mCherry]*	This study	GR3525
*C. elegans* strain GR3526*: gas-1(fc21); kynu-1(tm4924)*	This study	GR3526
*C. elegans* strain GR3527*: gas-1(fc21); kynu-1(tm4924); mgEx882[Prpl-28::cmtr-1(G126R) cDNA::GFP + myo-2::mCherry]*	This study	GR3527
*C. elegans* strain GR3533*: srrt-1(mg781[G310E]); unc-119(ed3); mgTi66[Pcmtr-1::cmtr-1 cDNA::GFP + unc-119(+)]*	This study	GR3533
*C. elegans* strain GR3534*: srrt-1(mg781[G310E]); unc-119(ed3); mgTi65[Pcmtr-1::cmtr-1(no G-patch) cDNA::GFP + unc-119(+)]*	This study	GR3534
*C. elegans* strain N2: wild type	CGC	N2
*C. elegans* strain GR3584*: him-5(e1490) oxTi405 V; gas-1(fc21) X*	This study	GR3584
*C. elegans* strain GR3585*: mgEx882[Prpl-28::cmtr-1(G126R) cDNA::GFP + myo-2::mCherry]*	This study	GR3585
*C. elegans* strain GR3586*: mgEx883[Prpl-28::cmtr-1(G126R) cDNA::GFP + myo-2::mCherry]*	This study	GR3586
*C. elegans* strain GR3587*: mgEx890[Prpl-28::cmtr-1(Delta11–42 G126R) cDNA::GFP + myo-2::mCherry]*	This study	GR3587
*C. elegans* strain GR3588*: mgEx899[Prpl-28::cmtr-1(Delta11–42 G126R) cDNA::GFP + myo-2::mCherry]*	This study	GR3588
*C. elegans* strain GR3589*: mgEx900[Prpl-28::cmtr-1(Delta11–42 G126R) cDNA::GFP + myo-2::mCherry]*	This study	GR3589
*C. elegans* strain GR3590*: gas-1(fc21); mgEx901[Prpl-28::cmtr-1(Delta11–42 G126R) cDNA::GFP + myo-2::mCherry]*	This study	GR3590
*C. elegans* strain GR3591*: gas-1(fc21); mgEx902[Prpl-28::cmtr-1(Delta11–42 G126R) cDNA::GFP + myo-2::mCherry]*	This study	GR3591
*C. elegans* strain GR3592*: gas-1(fc21); mgTi65[Pcmtr-1::cmtr-1(DeltaG-patch) cDNA::GFP + unc-119(+)]*	This study	GR3592
*C. elegans* strain GR3593*: gas-1(fc21); mgTi66[Pcmtr-1::cmtr-1 cDNA::GFP + unc-119(+)]*	This study	GR3593
*C. elegans* strain GR3594*: gas-1(fc21); mgEx891[Pnduf-2.2(3 kb)::gfp + myo-2::mCherry]*	This study	GR3594
*C. elegans* strain GR3595*: srrt-1(mg781[G310E]) I*	This study	GR3595
*C. elegans* strain GR3596*: srrt-1(mg781[G310E]); gas-1(fc21); mgEx903[Prpl-28::srrt-1 cDNA 50 ng/μl]*	This study	GR3596
*C. elegans* strain GR3597*: srrt-1(mg781[G310E]); gas-1(fc21); mgEx904[Prpl-28::srrt-1 cDNA 50 ng/μl]*	This study	GR3597
*C. elegans* strain GR3598*: srrt-1(mg781[G310E]); gas-1(fc21); mgEx905[Prpl-28::srrt-1 cDNA 50 ng/μl]*	This study	GR3598
*C. elegans* strain GR3599*: mgEx906[Prpl-28::srrt-1 cDNA 50 ng/μl]*	This study	GR3599
*C. elegans* strain GR3600*: mgEx907[Prpl-28::srrt-1 cDNA 50 ng/μl]*	This study	GR3600
*C. elegans* strain GR3601*: mgEx908[Prpl-28::srrt-1 cDNA 50 ng/μl]*	This study	GR3601
*C. elegans* strain GR3602*: mgEx909[Prpl-28::srrt-1 cDNA 50 ng/μl]*	This study	GR3602
Oligonucleotides		
*rps-23* qPCR Forward: aaggctcacattggaactcg	This study	N/A
*rps-23* qPCR Reverse: aggctgcttagcttcgacac	This study	N/A
*nduf-2.2* qPCR Forward: TGAAGTTTCCCGCTCGTATC	This study	N/A
*nduf-2.2* qPCR Reverse: TTCCACTTCCACGAACCATC	This study	N/A

## Highlights

- *cmtr-1* G-patch mutations activate broad expression of a neuronal complex I subunit
- *NDUFS2/nduf-2.2* is normally restricted to the dopaminergic neurons in *C. elegans*
- G-patch loss causes ectopic localization of CMTR-1 protein to Processing bodies
- Mutations in the mRNA binding protein Serrate phenocopy CMTR-1 G-patch mutations
