# Supplementary material for: CMTR-1 RNA methyltransferase mutations activate widespread expression of a dopaminergic neuron-specific mitochondrial complex I gene
Source: Curr Biol. Author manuscript; Available in PMC 2024 Jul 23. (PMC11265314; doi:10.1016/j.cub.2024.04.079)
Supplement: 2 [file NIHMS1993363-supplement-2.pdf]

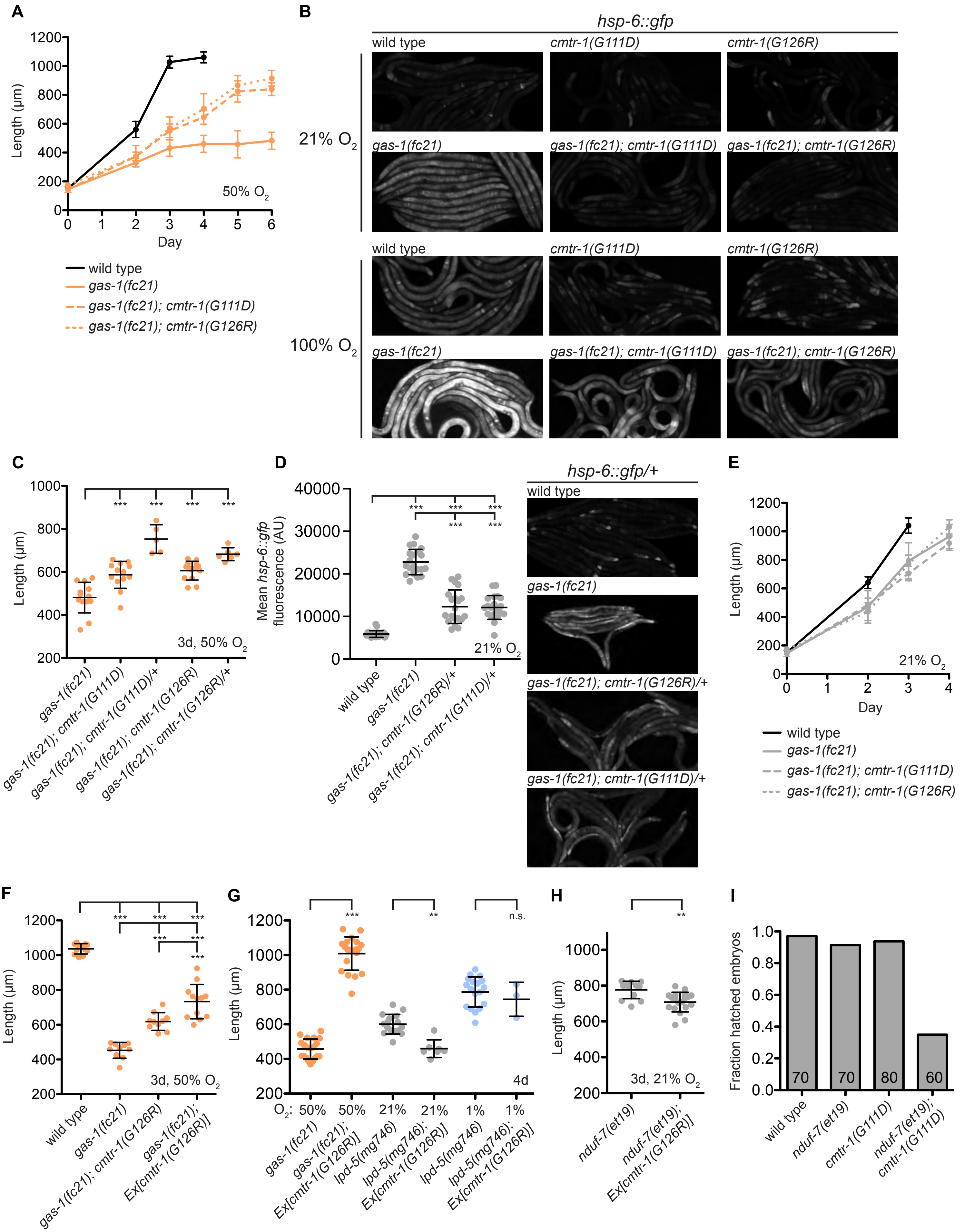

A
B
C
D
E
F
G
H
I
Length (µm)
Day
50% O2
wild type
gas-1(fc21)
gas-1(fc21); cmtr-1(G111D)
gas-1(fc21); cmtr-1(G126R)
hsp-6::gfp
cmtr-1(G111D)
cmtr-1(G126R)
21% O2
100% O2
gas-1(fc21); cmtr-1(G111D)
gas-1(fc21); cmtr-1(G126R)
3d, 50% O2
gas-1(fc21); cmtr-1(G111D)/+
gas-1(fc21); cmtr-1(G126R)/+
Mean hsp-6::gfp fluorescence (AU)
hsp-6::gfp/+
gas-1(fc21); Ex[cmtr-1(G126R)]
lpd-5(mg746)
lpd-5(mg746); Ex[cmtr-1(G126R)]
4d
n.s.
nduf-7(et19)
nduf-7(et19); Ex[cmtr-1(G126R)]
3d, 21% O2
Fraction hatched embryos
nduf-7(et19); cmtr-1(G111D)
70
70
80
60

Figure S1. CMTR-1 G-patch mutations restore health of *NDUFS2/gas-1(fc21)* mutants, related to Figure 1

A. Growth curve of wild type, *gas-1(fc21),* and *gas-1(fc21); cmtr-1* G-patch mutants exposed to continuous 50% oxygen. B. Fluorescent images of L4 animals carrying the *hsp-6::gfp* reporter incubated at 21% or 100% oxygen for 1 day at 20˚C. Exposure time = 100 ms, magnification = 69x. C. Growth of animals following 3 days exposure to 50% oxygen. D. Mean intestinal fluorescence (left) and fluorescent images (right) of the mitochondrial stress reporter *hsp-6::gfp* in L4 stage animals incubated at 21% oxygen. To facilitate the experiment, all strains are heterozygous for *hsp-6::gfp, him-5,* and a red fluorescent marker. Exposure time = 1000 ms, magnification = 69x. E. Growth curve of wild type, *gas-1(fc21),* and *gas-1(fc21); cmtr-1* mutants exposed to continuous 21% oxygen. F. Growth of animals following 3 days exposure to 50% oxygen. G. Growth of animals following 4 days exposure to 50%, 21%, or 1% oxygen. H. Growth of animals following 3 days exposure to 21% oxygen. I. Fraction of embryos laid that ultimately hatched. Labels correspond to number of embryos analyzed. For all panels statistical significance was calculated using one-way ANOVA followed by Tukey's Multiple Comparison Test. Error bars represent standard deviation. n.s. = not significant, * = p value <0.05, ** = p value <0.01, *** = p value <0.001.

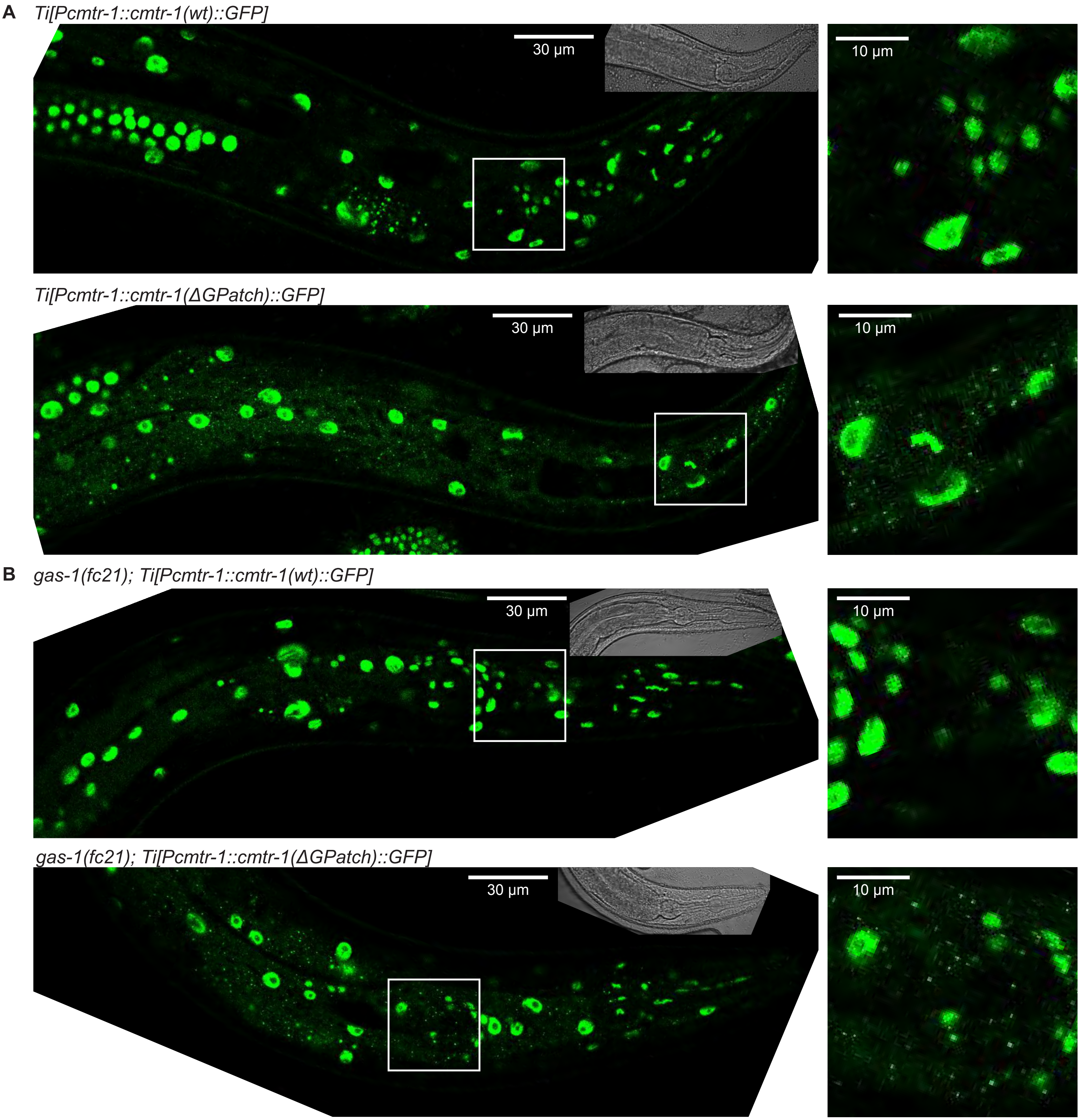

C

>*C. elegans* CMTR-1

```
MADRKSDEGEDEYQHKEQMVTNRTSSFQPKSTEDSISKLAKMRAADRREE
FMEERASFSAVKRGYQAGDDEEDDFTAEEEPPAKKPLTVAERLMAAMGHK
AGEGLGKHGQGISEPIASSTQRGRTGLGHNAGKATARDFNEVWDETTEEK
TVVERVEWMTDIEEEKRAEICEQLKDDKWMVIGKEKRTIDDETKFCSQQS
ITEMIEAKNVFDLMSDKDLREARTRANPYETIGSAFFQNRAAMKTANMDK
IYDWILSRENTENDRFLLKNPLQESQTAENVDRSEDLFYFADVCAGP...
```

>*H. sapiens* CMTR1

```
MKRRTDPECTAPIKKQKKRVAELALSLSSTSDDEPPSSVSHGAKASTTSL
SGSDSETEGKQHSSDSFDDAFKADSLVEGTSSRYSMYNSVSQKLMAKMGF
REGEGLGKYSQGRKDIVEASSQKGRRGLGLTLRGFDQELNVDWRDEPEPS
ACEQVSWFPECTTEIPDTQEMSDWMVVGKRKMIIEDETEFCGEELLHSVL
QCKSVFDVLDGEEMRRARTRANPYEMIRGVFFLNRAAMKMANMDFVFDRM
FTNPRDSYGKPLVKDREAELLYFADVCAGPGGFSEYVLWRKKWHAKG...
```

Nuclear Localization Sequence
Intrinsically Disordered Domain
G-patch Domain

Figure S2. G-patch mutant CMTR-1 is ectopically localized to P-bodies, which is necessary for its rescue of *gas-1(fc21)*, related to Figure 2

A. Confocal microscopy of *cmtr-1(wt)::gfp* and *cmtr-1(ΔG-patch)::gfp* driven by the endogenous *Pcmtr-1* promoter. Brightfield image is inset, white box corresponds to enlarged image on right. B. Confocal microscopy of *cmtr-1(wt)::gfp* and *cmtr-1(ΔG-patch)::gfp* driven by the endogenous *Pcmtr-1* promoter in the *gas-1(fc21)* mutant background. Brightfield image is inset, white box corresponds to enlarged image on right. C. Protein sequences of *C. elegans* CMTR-1 and human CMTR1 annotated with predicted nuclear localization signals (using cNLS mapper[S1]) and intrinsically disordered domains (using both DEPICTER[S2] and PrDOS[S3]).

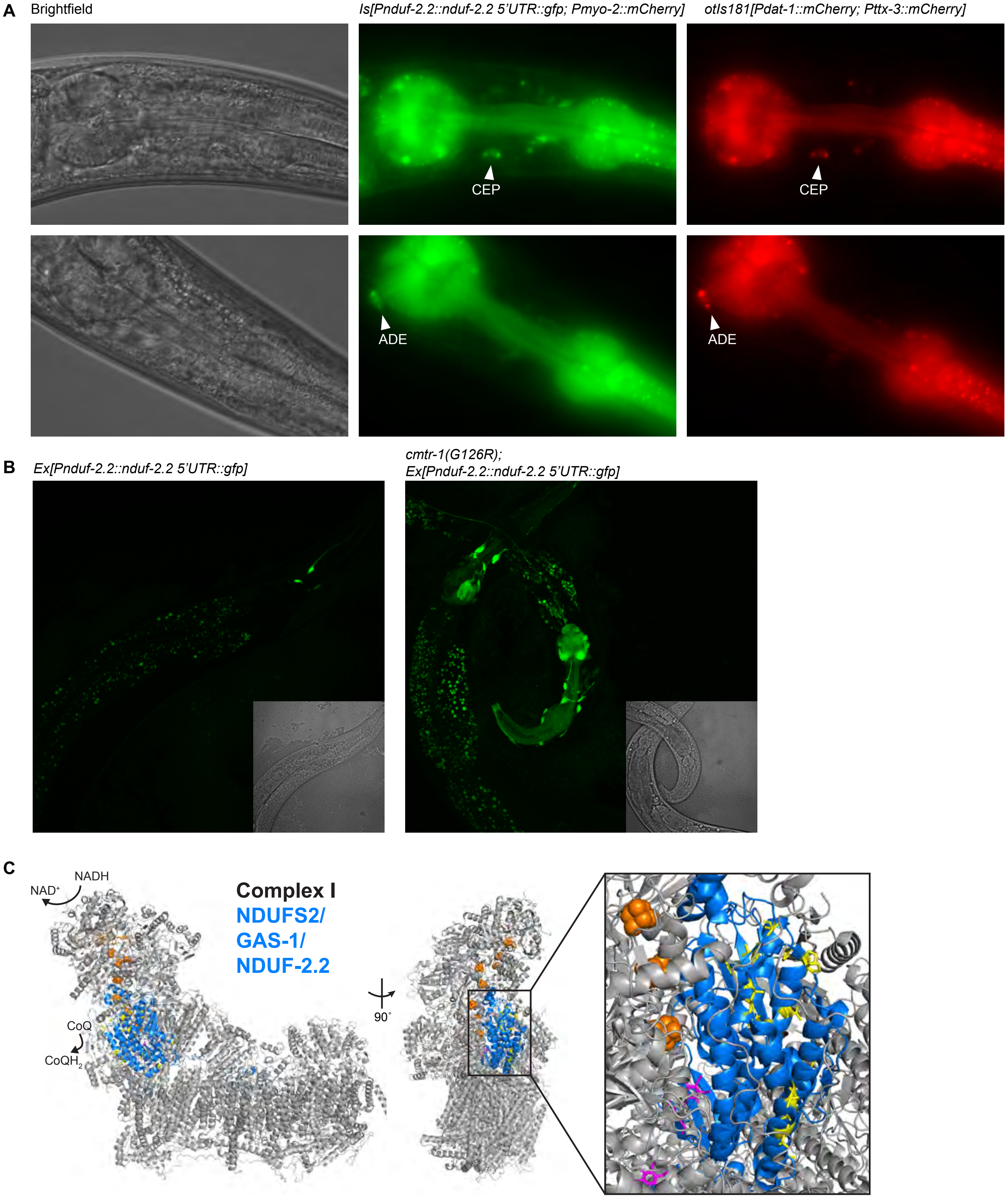
A
Brightfield
Is[Pnduf-2.2::nduf-2.2 5'UTR::gfp; Pmyo-2::mCherry]
otIs181[Pdat-1::mCherry; Pttx-3::mCherry]
CEP
CEP
ADE
ADE
B
Ex[Pnduf-2.2::nduf-2.2 5'UTR::gfp]
cmtr-1(G126R);
Ex[Pnduf-2.2::nduf-2.2 5'UTR::gfp]
C
NADH
NAD+
CoQ
CoQH2
Complex I
NDUFS2/
GAS-1/
NDUF-2.2
90˚

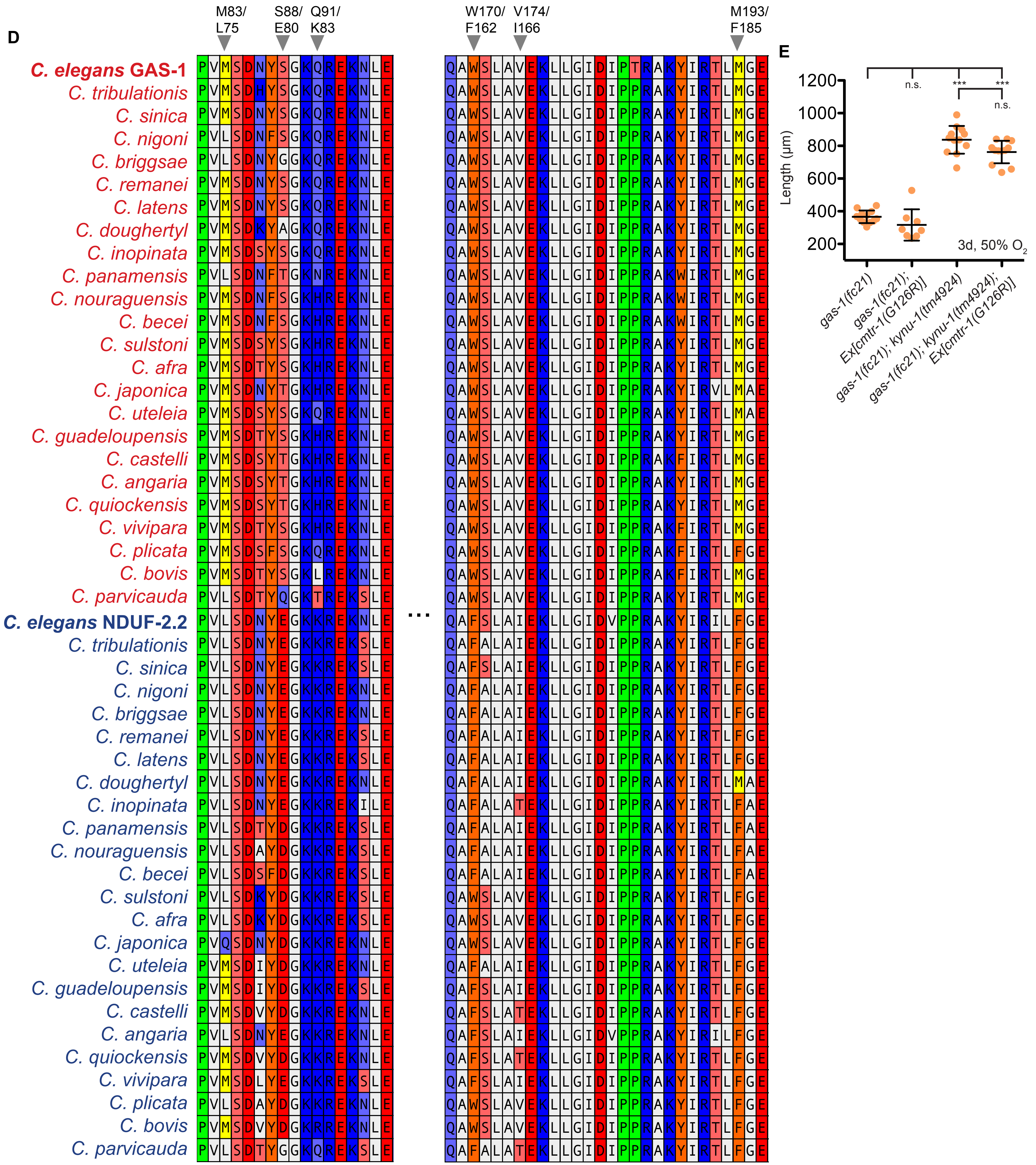
D
M83/ L75
S88/ E80
Q91/ K83
W170/ F162
V174/ I166
M193/ F185
C. elegans GAS-1
C. tribulationis
C. sinica
C. nigoni
C. briggsae
C. remanei
C. latens
C. doughertyl
C. inopinata
C. panamensis
C. nouraguensis
C. becei
C. sulstoni
C. afra
C. japonica
C. uteleia
C. guadeloupensis
C. castelli
C. angaria
C. quiockensis
C. vivipara
C. plicata
C. bovis
C. parvicauda
C. elegans NDUF-2.2
C. tribulationis
C. sinica
C. nigoni
C. briggsae
C. remanei
C. latens
C. doughertyl
C. inopinata
C. panamensis
C. nouraguensis
C. becei
C. sulstoni
C. afra
C. japonica
C. uteleia
C. guadeloupensis
C. castelli
C. angaria
C. quiockensis
C. vivipara
C. plicata
C. bovis
C. parvicauda
E
Length (μm)
1200
1000
800
600
400
200
n.s.
***
***
n.s.
3d, 50% O2
gas-1(fc21)
gas-1(fc21); Ex[cmtr-1(G126R)]
gas-1(fc21); kynu-1(tm4924)
gas-1(fc21); kynu-1(tm4924); Ex[cmtr-1(G126R)]

Figure S3. G-patch mutant CMTR-1 rescues *NDUFS2/gas-1(fc21)* by activating expression of the paralog NDUF-2.2, related to Figure 3

A. Compound microscopy of *nduf-2.2::gfp* co-localization in wild type with the dopaminergic neuronal marker *dat-1::mCherry*. White arrows correspond to dopaminergic CEP and ADE neurons. B. Confocal microscopy of *nduf-2.2::gfp* reporter in wild-type and *cmtr-1(G126R)* adult animals. C. Ovine complex I (PDB: 6ZKC[S4]) in closed conformation. NDUFS2 subunit is colored blue and the residues homologous to *C. elegans* residues that are divergent between paralogs GAS-1 and NDUF-2.2 are colored in yellow. They cluster on the surface-exposed side of NDUFS2, not the side close to Fe-S clusters (orange) and ubiquinone (purple). D. Multiple sequence alignment of *NDUFS2* homologs from *Caenorhabditis* species. Sequences were obtained from https://caenorhabditis.org/[S5] and alignments made with ClustalW. All species contain a GAS-1-like protein (red) and NDUF-2.2-like protein (blue). Labelled residues correspond to positions in *gas-1/nduf-2.2.* E. Growth of animals following 3 days exposure to 50% oxygen. Statistical significance was calculated using one-way ANOVA followed by Tukey's Multiple Comparison Test. Error bars represent standard deviation. n.s. = not significant, * = p value <0.05, ** = p value <0.01, *** = p value <0.001.

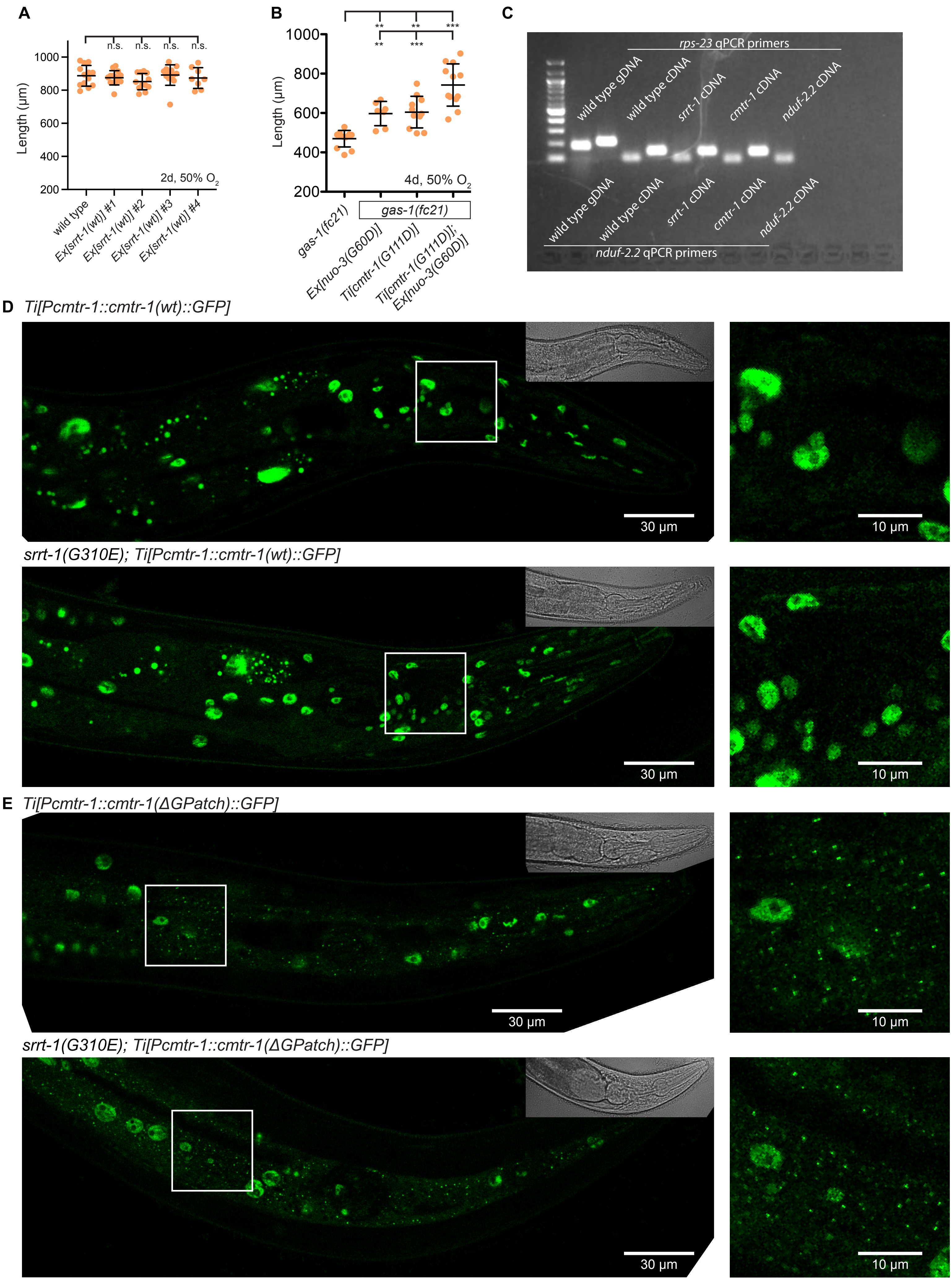
A
n.s.
n.s.
n.s.
n.s.
1000
800
600
400
200
Length (μm)
2d, 50% O2
wild type
Ex[srrt-1(wt)] #1
Ex[srrt-1(wt)] #2
Ex[srrt-1(wt)] #3
Ex[srrt-1(wt)] #4
B
**
**
***
**
***
1000
800
600
400
200
Length (μm)
4d, 50% O2
gas-1(fc21)
gas-1(fc21)
Ex[nuo-3(G60D)]
Ti[cmtr-1(G111D)]
Ti[cmtr-1(G111D)]; Ex[nuo-3(G60D)]
C
rps-23 qPCR primers
wild type gDNA
wild type cDNA
srrt-1 cDNA
cmtr-1 cDNA
nduf-2.2 cDNA
wild type gDNA
wild type cDNA
srrt-1 cDNA
cmtr-1 cDNA
nduf-2.2 cDNA
nduf-2.2 qPCR primers
D Ti[Pcmtr-1::cmtr-1(wt)::GFP]
30 μm
10 μm
srrt-1(G310E); Ti[Pcmtr-1::cmtr-1(wt)::GFP]
30 μm
10 μm
E Ti[Pcmtr-1::cmtr-1(ΔGPatch)::GFP]
30 μm
10 μm
srrt-1(G310E); Ti[Pcmtr-1::cmtr-1(ΔGPatch)::GFP]
30 μm
10 μm

Figure S4. Mutation of the RNA binding protein Serrate activates *nduf-2.2* and rescues *gas-1(fc21)*, related to Figure 4

A-B. Growth of animals following 2 days (A) or 4 days (B) exposure to 50% oxygen. C. Non-quantitative PCR with primers amplifying *nduf-2.2* or *rps-23* demonstrating that spliced cDNA is being amplified and that *nduf-2.2* primers do not amplify any DNA in the *nduf-2.2(ok437)* mutant. D-E. Confocal microscopy of either *cmtr-1(wt)::gfp* (D) or *cmtr-1(ΔG-patch)::gfp* (E) driven by the endogenous *Pcmtr-1* promoter in either wild-type or *srrt-1(G310E)* animals. Brightfield image is inset, white box corresponds to enlarged image on right.

NDUFS2/gas-1(fc21) **suppressor screen in hyperoxia**			
C. elegans **Mutation**	**Alleles**	**Human Homolog**	**Description**
cmtr-1(M97I)	2	CMTR1(M98)	mRNA Cap1 2'-O-methyltransferase
cmtr-1(M97T)	1	CMTR1(M98)	mRNA Cap1 2'-O-methyltransferase
cmtr-1(G106E)	3	CMTR1(G107)	mRNA Cap1 2'-O-methyltransferase
cmtr-1(G106R)	1	CMTR1(G107)	mRNA Cap1 2'-O-methyltransferase
cmtr-1(G111D)	2	CMTR1(G112)	mRNA Cap1 2'-O-methyltransferase
cmtr-1(G111S)	1	CMTR1(G112)	mRNA Cap1 2'-O-methyltransferase
cmtr-1(G126R)	2	CMTR1(G127)	mRNA Cap1 2'-O-methyltransferase
cmtr-1(G126E)	1	CMTR1(G127)	mRNA Cap1 2'-O-methyltransferase
srrt-1(G310E)	1	SRRT(G465)	Serrate RNA effector molecule
srrt-1(R497H)	2	SRRT(R646)	Serrate RNA effector molecule

Table S1. List of mutations reported in this study isolated from genetic screens, related to Figure 1

Through whole genome sequencing of mutant isolates we identified 13 independent alleles of CMTR-1 and three independent alleles of SRRT-1. The classical *gas-1(fc21)* suppressors *seg-1* and *seg-2* [S6] both carry *cmtr-1(G126E)* mutations (personal communication, Philip Morgan and Margaret Sedensky).

Supplemental References

S1. Kosugi, S., Hasebe, M., Tomita, M., and Yanagawa, H. (2009). Systematic identification of cell cycle-dependent yeast nucleocytoplasmic shuttling proteins by prediction of composite motifs. Proc National Acad Sci *106*, 10171–10176. 10.1073/pnas.0900604106.

S2. Barik, A., Katuwawala, A., Hanson, J., Paliwal, K., Zhou, Y., and Kurgan, L. (2020). DEPICTER: Intrinsic Disorder and Disorder Function Prediction Server. J Mol Biol *432*, 3379–3387. 10.1016/j.jmb.2019.12.030.

S3. Ishida, T., and Kinoshita, K. (2007). PrDOS: prediction of disordered protein regions from amino acid sequence. Nucleic Acids Res *35*, W460–W464. 10.1093/nar/gkm363.

S4. Kampjut, D., and Sazanov, L.A. (2020). The coupling mechanism of mammalian respiratory complex I. Sci New York N Y *370*. 10.1126/science.abc4209.

S5. Stevens, L., Rooke, S., Falzon, L.C., Machuka, E.M., Momanyi, K., Murungi, M.K., Njoroge, S.M., Odinga, C.O., Ogendo, A., Ogola, J., et al. (2020). The Genome of Caenorhabditis bovis. Curr Biol *30*, 1023-1031.e4. 10.1016/j.cub.2020.01.074.

S6. Kayser, E.-B., Sedensky, M.M., and Morgan, P.G. (2004). The effects of complex I function and oxidative damage on lifespan and anesthetic sensitivity in Caenorhabditis elegans. Mechanisms of Ageing and Development 125, 455–464. 10.1016/j.mad.2004.04.002.
